# Remarks on Fractures of the Scapula

**Published:** 1857-09

**Authors:** 


*Remarks on Fractures of the Scapula, with cases presenting striking peculiarities; read before the Medical Society of the State of Georgia, April, 1857. By L. A. Dugas, M. D., and Professor of Surgery.*

This is a well written and interesting monograph of 22 pages, and to the surgeon especially it will well repay for a careful perusal.

